# Influence of Strain Rate on the Strain-Induced Martensite Transformation in Austenitic Steel AISI 321 and Barkhausen Noise Emission

**DOI:** 10.3390/ma18153714

**Published:** 2025-08-07

**Authors:** Mária Čilliková, Nikolaj Ganev, Ján Moravec, Anna Mičietová, Miroslav Neslušan, Peter Minárik

**Affiliations:** 1 Faculty of Mechanical Engineering, University of Žilina, Univerzitná 1, 010 26 Žilina, Slovakia; maria.cillikova@fstroj.uniza.sk (M.Č.); jan.moravec@fstroj.uniza.sk (J.M.); anna.micietova@fstroj.uniza.sk (A.M.); miroslav.neslusan@fstroj.uniza.sk (M.N.); 2 Faculty of Nuclear Sciences and Physical Engineering, Czech Technical University in Prague, Trojanova 13, 12 000 Prague, Czech Republic; nikolaj.ganev@fjfi.cvut.cz; 3 Center of Competence “Smart Mechatronic, Eco- and Energy-Saving Systems and Technologies”, Technical University of Gabrovo, 5300 Gabrovo, Bulgaria; 4 Research Centre, University of Žilina, Univerzitná 1, 010 26 Žilina, Slovakia; 5 Faculty of Mathematics and Physics, Charles University, Ke Karlovu 5, 121 16 Prague, Czech Republic

**Keywords:** Barkhausen noise, austenitic steel, martensite, tensile test

## Abstract

This study investigates the evolution of strain-induced martensite (SIM) and its effect on magnetic Barkhausen noise (MBN) in AISI 321 austenitic stainless steel subjected to uniaxial tensile testing. Using X-ray diffraction and the Barkhausen noise technique, the formation and distribution of SIM were analysed as functions of plastic strain and strain rate. The results show that MBN is primarily governed by plastic deformation and strain rate rather than residual stress. The martensite fraction increases from 10% at low strains to 42.5% at high strains; however, accelerated strain rates significantly reduce martensite formation to approximately 25%. The increase in martensite density enhances the magnetic exchange interactions among neighbouring islands, resulting in stronger and more numerous MBN pulses. The anisotropy of MBN is also influenced by the initial crystallographic texture of the austenite. These findings highlight the strong correlation between MBN and SIM evolution, establishing MBN as a sensitive, non-destructive tool for assessing martensitic transformation and optimising deformation parameters in austenitic steels.

## 1. Introduction

Austenitic steels are widely used in applications that demand a combination of high corrosion resistance, strength, and ductility. The stability of the austenitic phase at room temperature is primarily governed by alloying elements, such as nickel (Ni) and manganese (Mn), both known as austenite stabilisers [1]. In contrast, chromium (Cr) and molybdenum (Mo) act as ferrite stabilisers but also significantly enhance corrosion resistance. Due to their high alloy content, austenitic steels are susceptible to strain-induced martensitic transformation when subjected to various processing techniques, such as forming, machining, or shot-peening [2,3,4,5]. This transformation can adversely affect the functional performance of components in service. Therefore, monitoring the extent of the strain-induced transformation and identifying the influencing factors are crucial.

Talonen et al. [6] provided an extensive overview of techniques suitable for assessing strain-induced martensite, including X-ray diffraction (XRD), Satmagan, density measurements, optical methods, and magnetic balance. Among these, magnetic methods offer a promising approach due to the strong magnetic contrast between non-ferromagnetic austenite and ferromagnetic martensite. Consequently, magnetic Barkhausen noise (MBN) emissions have been utilised for this purpose. Haušild et al. [7] employed various techniques, including magnetic measurements, to confirm the presence and quantify the amount of strain-induced martensite in AISI 301 austenitic stainless steel. In a subsequent study [8], they applied the MBN technique under biaxial loading to monitor martensite fractions. Astudillo et al. [9] investigated MBN emissions in AISI 304 during uniaxial tensile testing up to fracture. Kleber and Barroso [10] demonstrated that MBN could characterise martensite formation in AISI 304L after shot-peening, showing a strong dependence on processing conditions. Tavares et al. [11] explored MBN in supermartensitic stainless steel to correlate martensite/austenite partitioning with hydrogen embrittlement-related failures.

MBN arises during cyclic magnetisation of ferromagnetic materials due to irreversible and discontinuous domain wall (DW) motion [10]. DWs interact with various microstructural obstacles, such as grain boundaries, dislocations, precipitates, and non-ferromagnetic phases. Hence, MBN signals comprise microstructure and stress state information, with pulse height and density reflecting the superposition of multiple influencing factors [12,13,14,15,16,17,18]. Traditionally, MBN techniques are used for surface evaluation, including monitoring thermal softening from grinding or surface hardening from mechanical processing [13,17,18]. MBN increases due to thermal softening or decreases with hardening, and these changes are compared to baseline measurements from untreated surfaces to assess the material conditions.

In contrast, using MBN to monitor strain-induced transformation in austenitic steel is one of this technique’s most sensitive applications. Bulk austenite does not emit MBN, aside from negligible background signals from the sensor or mechanical vibrations, which can be filtered or subtracted [19]. Therefore, MBN signals can be directly attributed to the presence and extent of martensite. This study investigates strain-induced transformation in AISI 321 stainless steel using the MBN technique, particularly emphasising the effects of plastic strain and strain rate.

## 2. Materials and Methods

**Figure 1:**
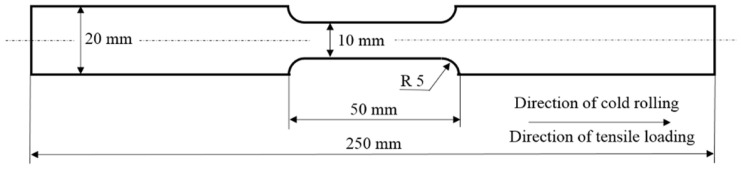
Illustration of the samples employed for the plastic straining; the length of the gauged region is *L*_0_ = 40 mm [20].

**Figure 2:**
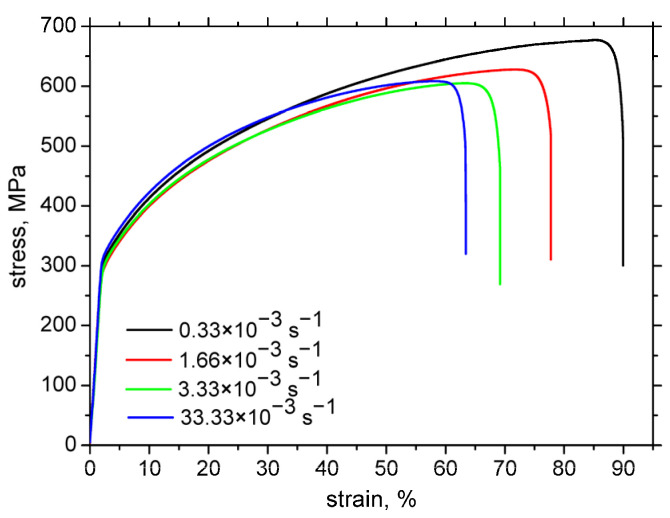
Engineering stress–strain (engineering) curves of AISI 321 as a function of the strain rate.

The experiments were conducted on cold-rolled AISI 321 austenitic stainless steel. Figure 1 shows samples prepared from a 2 mm thick sheet, aligned with the rolling direction. The chemical composition is listed in Table 1. The material’s mechanical properties include an ultimate tensile strength of 540–680 MPa, a yield strength of 202–220 MPa, and a hardness of 88 HRB. The engineering stress–strain curves (using the specimens as that depicted in Figure 1) are presented in Figure 2. It can be reported that the acceleration of strain rate results in a decrease of the ultimate strength and elongation at break.

The samples were subjected to tensile loading to achieve plastic strains of 5%, 25%, 35%, 45%, 55%, and 60% at four strain rates: 0.33 × 10^−3^, 1.66 × 10^−3^, 3.33 × 10^−3^, and 33.3 × 10^−3^ s^−1^. These deformation parameters were selected based on preliminary tests evaluating MBN response. To compare the MBN emissions at the same strains, the chosen plastic strains were limited up to 60% (55% for the highest strain rate of 33.3 × 10^−3^ s^−1^).

Hardness (*HV1*) measurements were carried out using an Innova Test 400TM tester under a 1 kg load applied for 10 s. Each result was averaged from five measurements. The temperature during tensile testing was monitored using a K-type thermocouple micro-welded at the specimen centre. The analogue signal was digitised and recorded at 100 Hz using the DasyLab 2016 software (version 14.2.0). Microstructural characterisation was performed using Electron Backscatter Diffraction (EBSD) in a ZEISS Auriga Compact SEM with an EDAX EBSD camera. The analysis was conducted using the TSL OIM 8 software. Image quality (IQ) maps were used to visualise the lattice defects and grain boundaries.

MBN measurements were taken post-deformation using a RollScan 350 system, with data processed in the MicroScan 500 software. The magnetising voltage and frequency were set at 16 V and 125 Hz, respectively. The S1-18-12-01 sensor was used to record MBN pulses in the 10–1000 kHz frequency range. MBN was measured along both the rolling direction (RD) and transverse direction (TD). The root-mean-square (RMS) value of the signal, peak position (PP) of the MBN envelope, and number of individual MBN pulses were all analysed.

Residual stress and phase analyses were performed with an X’Pert PRO MPD diffractometer with ω-geometry. Manganese and chromium radiation were used to measure lattice deformation from the {311} and {211} planes of the austenite and martensite phases, respectively. The sin^2^*ψ* method was used to calculate the residual stresses, with X-ray elastic constants of *s*_1_ = −1.2 TPa^−1^, *½s*_2_ = 7.18 TPa^−1^ for austenite, and *s*_1_ = −1.25 TPa^−1^, *½s*_2_ = 5.76 TPa^−1^ for martensite. Nine tilts of angles *ψ*, i.e., sin^2^*ψ* = 0; 0.15; …; 0,6 for positive and negative values of angles *ψ*. Phase fractions, crystallite size (coherently diffracting domains), and microstrain were determined using cobalt radiation and Rietveld refinement in the MStruct software (version 0.1) [21]. The experimental errors of each value are the standard deviations of the calculation of the given parameters.

## 3. Results

### 3.1. EBSD Observations

**Figure 3:**
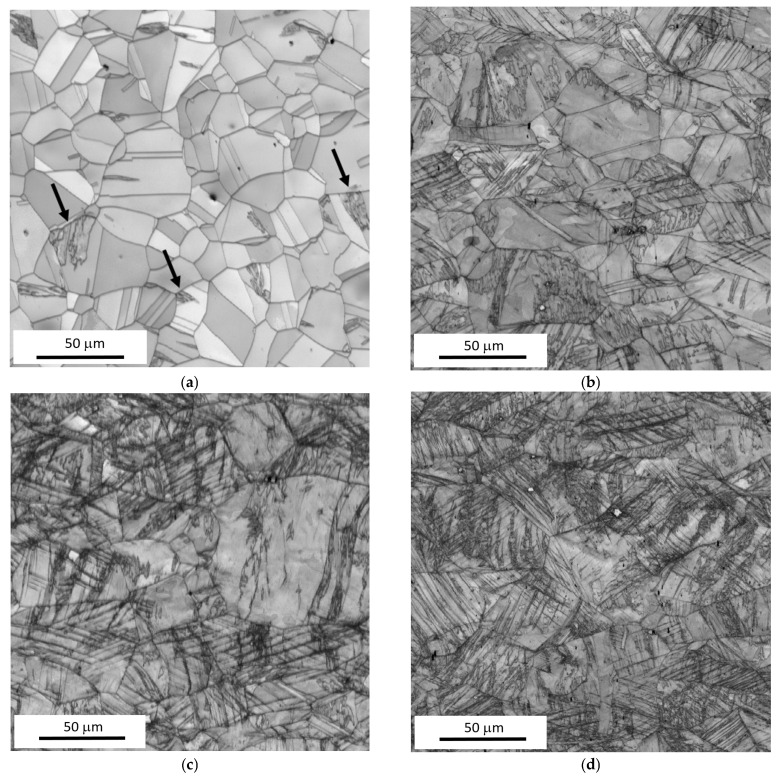
EBSD images (IQ) of samples at a strain rate of 0.33 × 10^−3^ s^−1^. (**a**) Bulk, (**b**) 25%, (**c**) 35%, and (**d**) 55%. The black arrows in (**a**) indicate martensite islands.

Figure 3a illustrates that the microstructure of AISI 321 stainless steel prior to tensile testing was not composed exclusively of equiaxed austenitic grains. Discrete, localised regions of strain-induced martensite were also present within the austenitic matrix. These martensitic islands are attributed to the cold rolling process, which is applied subsequently to annealing to enhance the surface smoothness. Additionally, Figure 3 indicates that plastic deformation during tensile testing promotes further transformation of austenite to strain-induced martensite. Both the volume fraction of martensite and the number of martensitic regions increased progressively with the plastic strain. This increase is associated with the enhanced formation and intersection of shear bands during strain hardening of the austenitic matrix. The intersections of these shear bands serve as preferential sites for martensitic transformation and subsequent volumetric growth of the martensite phase.

### 3.2. XRD and Hardness Measurements

**Figure 4:**
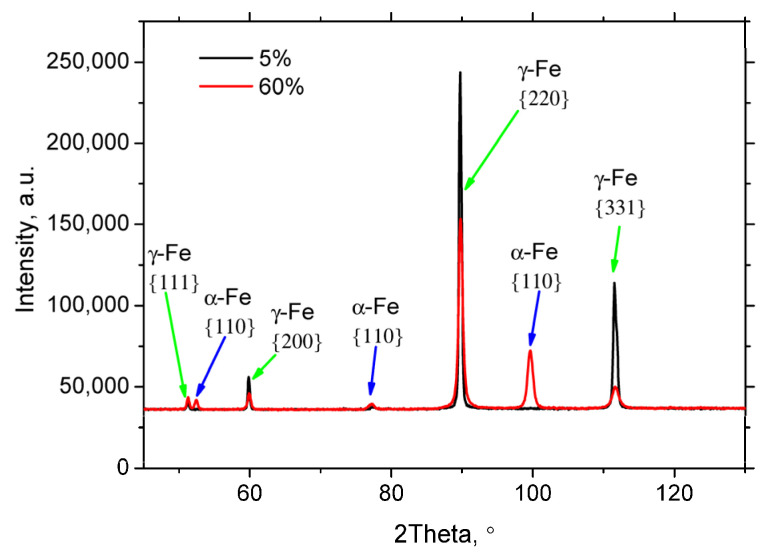
XRD diffraction patterns for a strain rate of 0.33 × 10^−3^ s^−1^.

**Figure 5:**
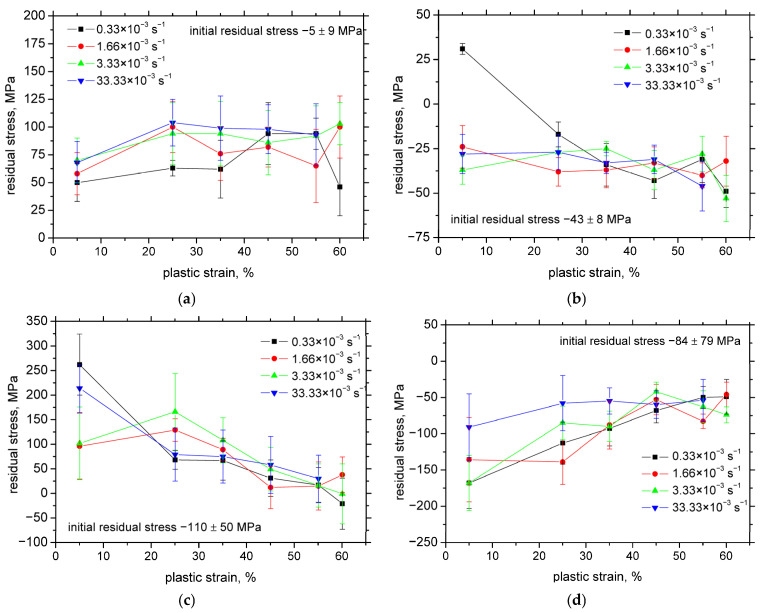
Residual stresses as a function of plastic strain. (**a**) RD in austenite; (**b**) TD in austenite; (**c**) RD in martensite; and (**d**) TD in martensite.

XRD diffraction patterns depicted in Figure 4 clearly prove the presence of the strain-induced martensite in the parental austenite matrix. Figure 5a,b reveal that the austenitic matrix initially exhibited low compressive residual strains in both the rolling direction (RD) and the transverse direction (TD).

Upon tensile loading along RD, a substantial increase in tensile residual stresses was observed in RD, which was counterbalanced by compressive residual stresses in TD during the initial 5% of plastic deformation. Beyond this point, residual stresses remained largely stable with continued straining, with the exception of a notable variation in TD between 5% and 25% strain at a strain rate of 0.33 × 10^−3^ s^−1^.

In contrast, Figure 5c,d illustrate that the evolution of residual stresses in the martensitic phase during deformation follows a slightly different trend. After 5% strain, the martensite phase exhibits relatively high tensile residual stress along RD and compressive stress along TD. With increasing the plastic deformation, these residual stresses gradually diminish in both directions. Moreover, Figure 5c,d indicate that the strain rate has only a minor influence on the development of residual stresses, as the stress magnitudes remain comparable across all tested strain rates, particularly at higher levels of plastic strain.

**Figure 6:**
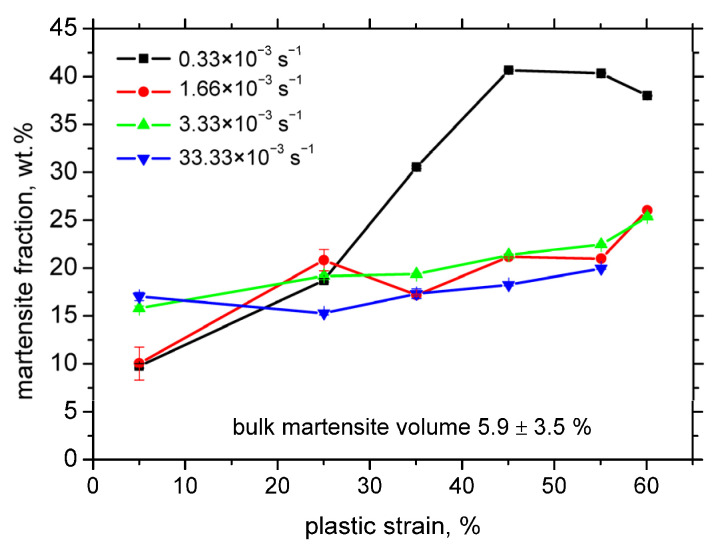
Fraction of strain-induced martensite as a function of plastic strain in RD (wt.%).

Figure 6 demonstrates that the evolution of the martensite fraction is strongly dependent on the applied strain rate, particularly distinguishing the lowest strain rate of 0.33 × 10^−3^ s^−1^ from the higher ones. At this lowest strain rate, a pronounced increase in the martensite fraction is observed following a 5% plastic deformation, continuing steadily up to 55% strain, where it reaches approximately 43%. In contrast, the samples deformed at higher strain rates exhibit only a modest and nearly uniform increase in martensite content beyond 5% of plastic deformation, regardless of the exact strain rate, culminating in a martensite fraction of approximately 25% after 60% plastic deformation.

**Figure 7:**
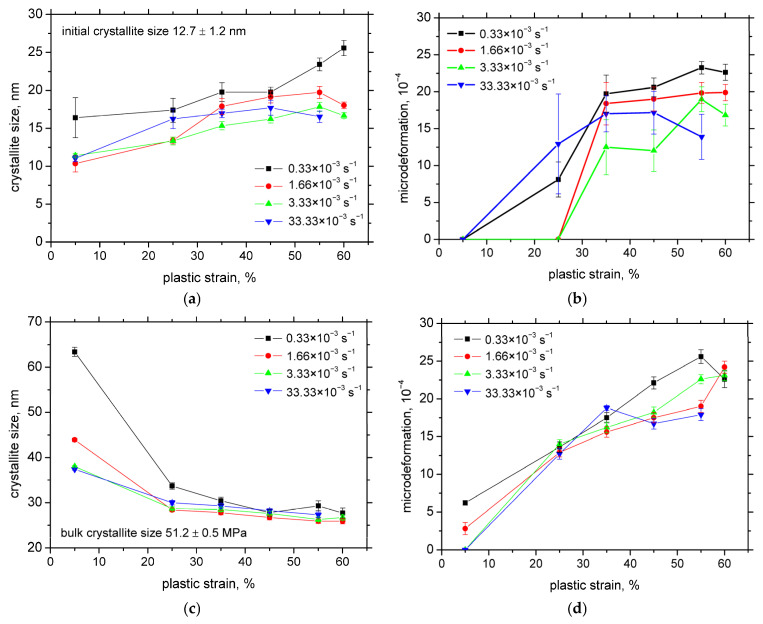
Crystallite size and microdeformation as a function of plastic strain (RD). (**a**) Crystallite size in martensite; (**b**) microdeformation in martensite; (**c**) crystallite size in austenite; and (**d**) microdeformation in austenite.

Figure 7a illustrates a moderate increase in the martensite crystallite size with the increasing plastic strain across all examined strain rates. Similar to the evolution of the martensite fraction, the crystallite size growth in the sample deformed at 0.33 × 10^−3^ s^−1^ displays a slightly different trend compared to those at higher strain rates. Figure 7b shows that the microdeformation initially increases with the strain but reaches saturation at relatively early stages. The differences in the microdeformation behaviour among the various strain rates are minimal, particularly at higher strains. Figure 7c,d also demonstrate that the strain hardening mechanism is a mixture of phase transformation and the superimposing contribution of austenite strain hardening due to a decreasing crystallite size (which indicates increasing dislocation density) as well as a growing microdeformation. The differences among the strain rates are only minor.

**Figure 8:**
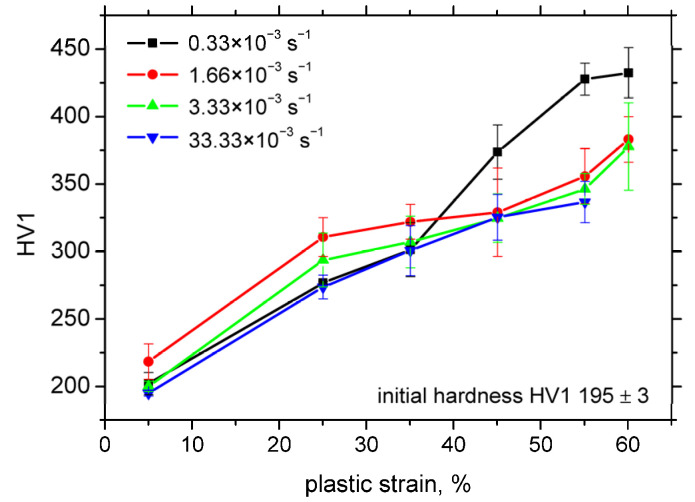
Hardness *HV1* as a function of plastic strain and strain rate.

The formation of strain-induced martensite also contributes significantly to the observed increase in hardness, as shown in Figure 8. This is primarily attributed to the increasing volume fraction of the harder martensitic phase replacing the softer austenitic matrix. The strain rate exerts only a minor influence on the hardness evolution; however, the sample deformed at the lowest strain rate again exhibits a distinct behaviour. In this case, the higher martensite content at elevated plastic strains results in correspondingly higher hardness values.

### 3.3. MBN Measurements

**Figure 9:**
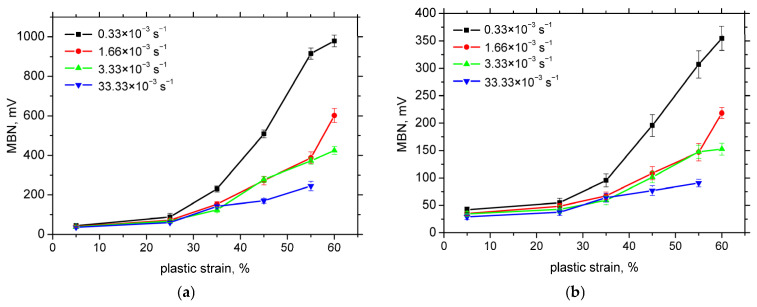
MBN as a function of plastic strain. (**a**) RD; (**b**) TD.

The first magnetic MBN pulses exceeding the background sensor noise were detected after 5% plastic strain in both RD and TD. Notably, no MBN signal indicating the presence of strain-induced martensite was observed in the undeformed samples despite the detection of a small amount of martensite by both XRD and EBSD. Figure 9 shows that the MBN intensity increased progressively with the plastic deformation without exhibiting saturation, even at the highest strain levels and lowest strain rates. This trend contrasts with the martensite fraction behaviour presented in Figure 6.

At higher plastic strains, more pronounced differences in MBN response were observed between the different strain rates. The highest MBN values for both RD and TD were recorded at the lowest strain rate of 0.33 × 10^−3^ s^−1^. The strain rates of 1.66 × 10^−3^ s^−1^ and 3.33 × 10^−3^ s^−1^ yielded nearly identical MBN responses (with the exception of the 60% strain level), but these were substantially lower than those for the lowest strain rate. A further moderate reduction in MBN was noted at the highest strain rate of 33.33 × 10^−3^ s^−1^. Additionally, Figure 9 reveals that MBN values in RD were consistently higher than those in TD, suggesting that domain walls in strain-induced martensite are preferentially aligned along RD, likely at the expense of TD.

**Figure 10:**
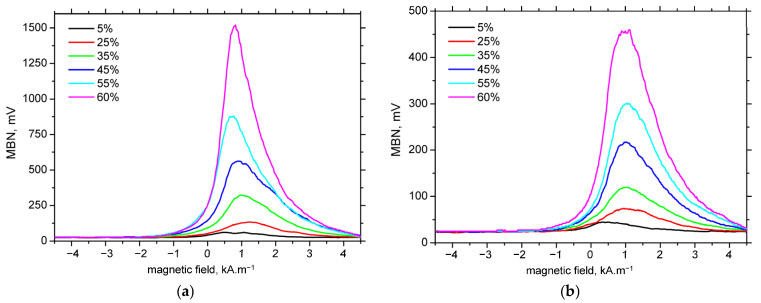
MBN envelopes as a function of plastic strain for a strain rate of 1.66 × 10^−3^ s^−1^. (**a**) RD; (**b**) TD.

**Figure 11:**
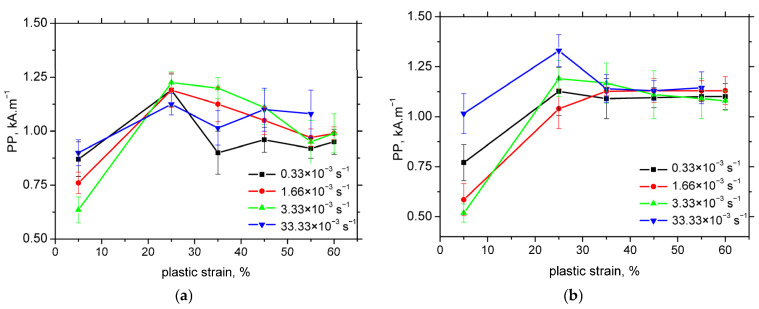
*PP* as a function of plastic strain. (**a**) RD; (**b**) TD.

The MBN envelopes and the corresponding peak positions (PP) are presented in Figure 10 and Figure 11, respectively. These figures demonstrate that at lower levels of plastic strain, the MBN envelopes are shifted towards higher magnetic field values, followed by early saturation at higher strains. The influence of strain rate on the envelope position and PP is negligible, with the exception of minor variations observed at the lowest plastic strains in the TD.

**Figure 12:**
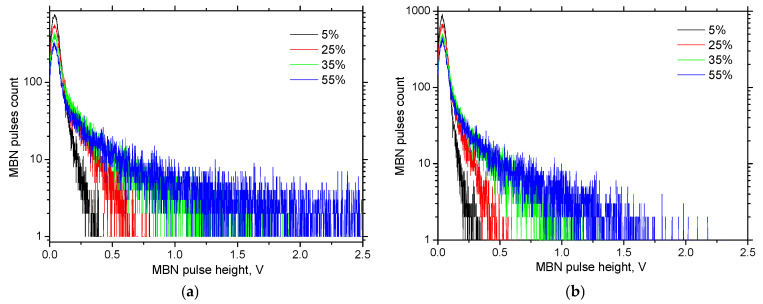
MBN pulse height distribution as a function of plastic strain (RD). (**a**) 0.33 × 10^−3^ s^−1^; (**b**) 33.33 × 10^−3^ s^−1^.

Figure 12 presents the modified distribution of MBN pulses as a function of pulse amplitude, revealing the evolution of magnetic activity with the increasing plastic strain. At low plastic strains, the distribution is dominated by a high number of low-amplitude MBN pulses, while pulses of higher amplitude are virtually absent. In contrast, at higher plastic strains, the number of low-amplitude pulses decreases, coinciding with a notable increase in higher-amplitude MBN pulses. This trend is observed in both RD and TD, suggesting a common underlying mechanism.

The shift toward higher-amplitude MBN pulses with increasing strain implies that certain martensite islands grow in size as plastic deformation progresses. Larger martensite islands are associated with an increased number and length of 180° DWs, which are recognised as primary sources of high-intensity MBN signals [22,23]. Moreover, Figure 12 indicates that the growth of martensite islands, and, hence, the number of strong MBN pulses, is suppressed at higher strain rates.

**Figure 13:**
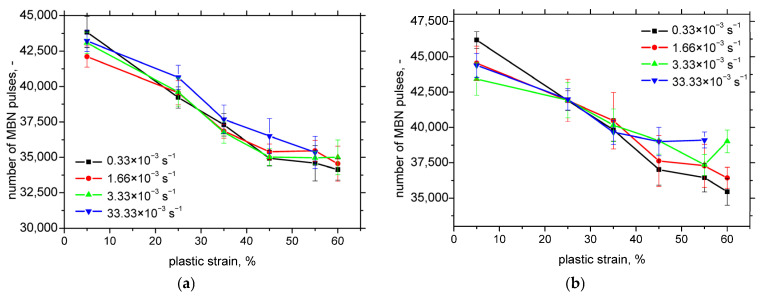
Number of MBN pulses as a function of plastic strain. (**a**) RD; (**b**) TD.

Finally, Figure 13 shows that the total number of detected MBN pulses decreases with the increasing plastic strain in both RD and TD. This suggests that the reduction in low-amplitude pulses outweighs the increase in stronger pulses at higher strain levels, leading to an overall decline in MBN activity.

## 4. Discussion of Obtained Results

The martensite volume fraction determined by XRD exhibits saturation at the highest levels of plastic strain for the lowest strain rate of 0.33 × 10^−3^ s^−1^. However, previous studies have shown that both light microscopy and EBSD techniques report a continued increase in martensite volume fractions up to 60% of plastic strain [20]. These techniques are limited by their relatively small sampling areas compared to XRD, which analyses a significantly larger irradiated volume. Nevertheless, the martensite fraction obtained via XRD at high plastic strains may be influenced by the preferential orientation of the martensite phase in the RD, as suggested by the MBN measurements. This is reasonable since DWs’ alignment generally follows the crystallographic orientation of the martensitic lattice [24].

**Figure 14:**
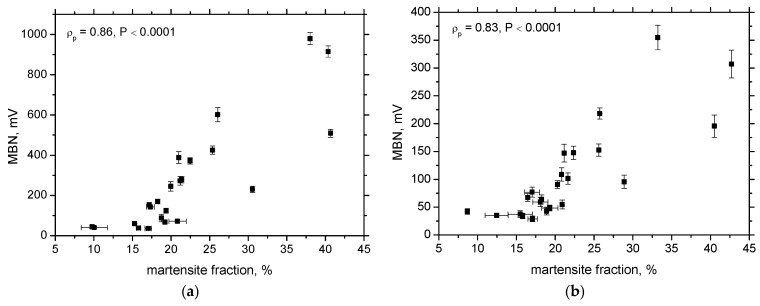
Correlation between martensite fraction and MBN. (**a**) RD; (**b**) TD.

In this context, the MBN technique offers a unique combination of advantages from the aforementioned methods. It provides rapid measurements and a large sensing area comparable to that of XRD and displays continuous signal intensity growth with increasing plastic strain in this specific case. Despite some discrepancies in the evolution trends, Figure 14 shows a good correlation between the MBN signal amplitude and the martensite volume fraction determined by XRD. The RD exhibits superior sensitivity, as reflected by the broader dynamic range of MBN values.

**Figure 15:**
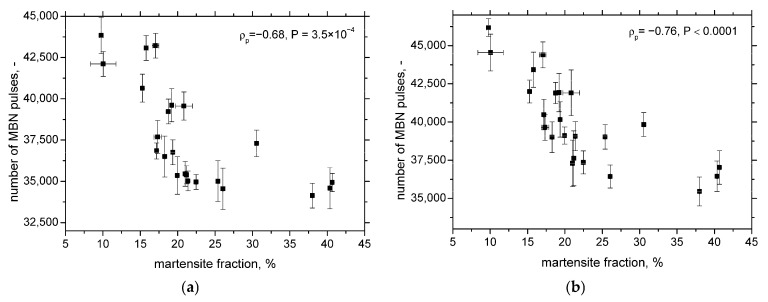
Correlation between martensite fraction and number of MBN pulses. (**a**) RD; (**b**) TD.

However, PP values derived from MBN measurements are unreliable indicators of martensite content due to their early saturation and significant overlap across different strain rates. In contrast, the total number of detected MBN pulses decreases with increasing the plastic strain and corresponding martensite fraction, as shown in Figure 15. While the correlation coefficients for this trend are somewhat lower than those in Figure 14, they remain sufficiently high to be considered acceptable.

The *rms* of the MBN signals is defined as follows [25]:

(1) $$rms=\sqrt{\frac{1}{n}\sum_{i=1}^{n}X_{i}^{2}}$$

where *n* is the total number of MBN pulses (events) captured at the specific frequency range, and *X_i_* is the amplitude of the individual pulses. The observed increase in MBN with the plastic strain and the corresponding martensite fraction, when expressed in terms of its root-mean-square (RMS) value, can be attributed to the following: (i) an increasing number of MBN pulses, (ii) an increase in the amplitude of individual pulses, or (iii) a combined increase in both the number and amplitude of pulses. However, when comparing the evolution of MBN and the number of MBN pulses shown in Figure 9, Figure 13 and Figure 15, it becomes evident that the number of MBN pulses actually decreases with increasing the plastic strain and martensite content. Therefore, the increase in MBN signal must primarily result from the growing amplitude of individual MBN pulses [26,27].

A comparison between Figure 5 and Figure 9 further supports the conclusion that residual stresses do not significantly influence MBN behaviour in this context. While MBN continues to increase with the strain, the tensile residual stresses in RD decrease. Likewise, although compressive stresses in TD decline with the strain, the lowest MBN values are observed at the highest strain rate (33.33 × 10^−3^ s^−1^), even at low plastic strains where the compressive stresses are minimal.

As shown in Figure 7, the martensite crystallite size increases only modestly with the plastic strain, an effect too small to account for the pronounced rise in MBN. Additionally, while increasing the microdeformation generally indicates a rise in the amplitude of local stress fields within the lattice, such conditions would typically lead to a reduction in MBN [28]—a trend not observed in this case. Hence, it can be concluded that the increase in MBN is governed primarily by the martensite fraction and the direction of applied tensile loading.

Unlike XRD, which detects similar martensite fractions in both RD and TD, the MBN technique is directionally sensitive. This highlights the importance of knowing the direction of applied stress when using MBN for in-service monitoring. Alternatively, the sensor should be rotated to detect the magnetic easy axis, typically aligned with RD [29].

It is also important to note that, in contrast to XRD and EBSD, the MBN technique cannot reliably detect martensite fractions below approximately 9% in AISI 321 austenitic steel. The first MBN pulses above background noise are typically observed only near this threshold.

**Figure 16:**
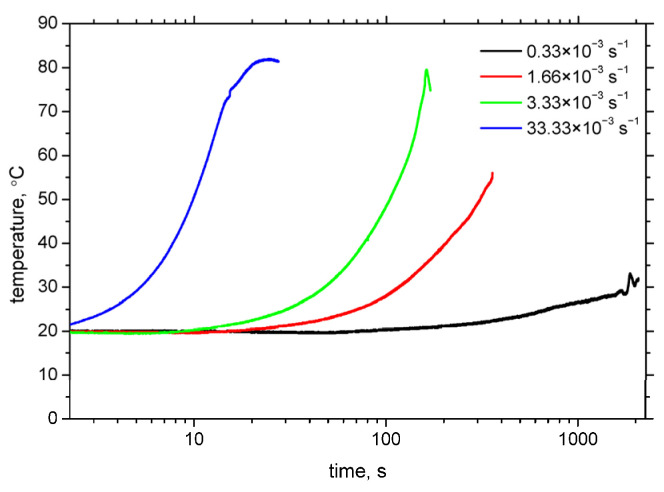
Evolution of temperature until sample breakage for the different strain rates.

Temperature rise during plastic deformation plays a significant role in the strain-induced martensitic transformation. Haušild et al. [7] identified three primary contributors to temperature increase: (i) conversion of plastic deformation energy to heat (approximately 90% efficiency), (ii) latent heat from the phase transformation, and (iii) heat dissipation via thermal conduction, particularly to the grips and regions outside the necked zone. While the first two effects contribute to heating, the third acts to remove heat. At low strain rates, heat dissipation dominates, resulting in nearly isothermal deformation. However, as the strain rate increases, heat generation surpasses dissipation, leading to significant temperature rises, as shown in Figure 16. Due to the inherently low thermal conductivity of austenitic steels [30], plastic deformation at higher strain rates is effectively adiabatic. Comparing Figure 6 and Figure 16, it is evident that increasing temperature stabilises the austenite phase, thereby suppressing the strain-induced transformation to martensite.

**Figure 17:**
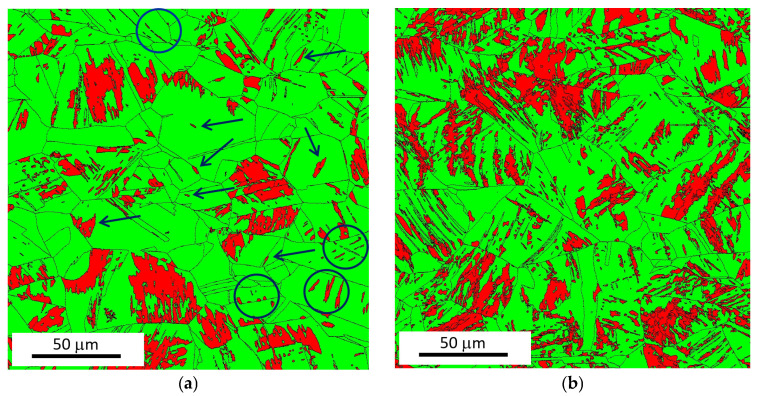
EBSD phase maps for a strain rate of 0.33 × 10^−3^ s^−1^. (**a**) Strain 25%; (**b**) strain 55%.

Plastic straining of austenite via dislocation glide (as evidenced by Figure 7c,d) plays a critical role in the formation of SIM. The accumulation of Shockley partial dislocations leads to the nucleation of SIM, particularly at dislocation intersections and grain boundaries [31,32]. As a result, SIM regions are preferentially found within austenite grains that have been strained beyond the yield point. Consequently, the microstructure consists of SIM-rich grains adjacent to others that remain largely unaffected by the phase transformation, especially at lower strain levels, as shown in Figure 17a. The density of SIM islands increases with progressive plastic straining, resulting in a higher overall SIM fraction. At the same time, the heterogeneity in SIM distribution decreases (see Figure 17b).

This evolution has a significant impact on magnetic MBN behaviour. A denser SIM network reduces the distance between neighbouring SIM islands, thereby enhancing magnetic exchange interactions [33]. When the SIM fraction is low, the matrix contains many isolated SIM islands and spots (highlighted by blue arrows and circles in Figure 17a), whose individual contributions to MBN are weak and often attenuated. The magnetisation process occurs in the form of avalanches [34]. When domain wall (DW) motion is initiated in a specific region, the interaction is propagated to adjacent regions. Shorter distances between SIM islands facilitate more effective and collective domain interactions, resulting in stronger MBN pulses and, thus, stronger overall MBN signals.

**Figure 18:**
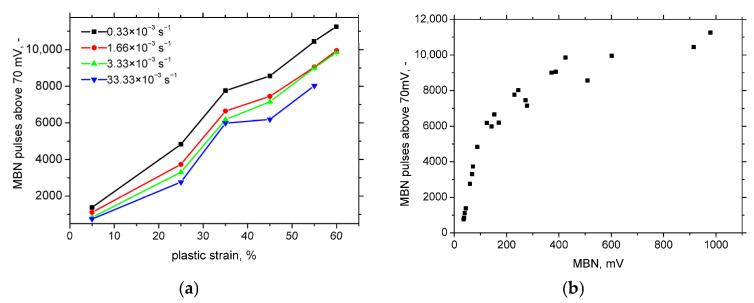
MBN pulses above 70 mV and their correlation against MBN. (**a**) MBN pulses above 70 mV; (**b**) MBN versus MBN pulses above 70 mV.

Figure 13 shows a decreasing total number of detected MBN pulses. However, the number of strong pulses—those exceeding the background noise level of approximately 70 mV—steadily increases with progressive plastic straining (see Figure 18a). In contrast, this number decreases as the strain rate increases. Each detected MBN pulse represents a collective motion of domain walls, but the actual number of domain walls involved is typically two to three orders of magnitude higher than the number of detected pulses [35]. As discussed earlier, SIM density and the associated magnetic exchange interactions are key factors influencing this behaviour.

Figure 18b confirms that MBN is predominantly governed by the strong MBN pulses. At lower MBN levels, the evolution is driven mainly by an increase in the number of strong pulses. However, beyond a certain point, this evolution saturates, and further increases in MBN are primarily due to the increased strength of the individual pulses exceeding a critical threshold at higher strain levels.

## 5. Conclusions

This study demonstrates that residual stress after tensile testing plays no significant role in magnetic Barkhausen noise (MBN). Instead, MBN is primarily governed by plastic strain and strain rate and should be interpreted in terms of martensite fraction and the mutual magnetic exchange interactions among neighbouring martensite islands. The martensite fraction increases from approximately 10% at low strains to 42.5% at higher strains. However, elevated strain rates significantly suppress martensite formation, reducing the fraction to about 25% even at high strains. The matrix hardens during uniaxial tension, and this hardening is largely due to the plastic deformation of austenite and martensitic transformation. For instance, the initial Vickers hardness (HV1) increases with the plastic strain from 200 to 425 for 5% and 60%, respectively.

At low strain rates, MBN in the rolling direction (RD) reaches up to 1000 mV at higher strains, while at higher strain rates, MBN remains below 600 mV. The initial crystallographic texture of the austenite, which is preferentially aligned along RD, also contributes to stronger MBN signals in RD compared to the transverse direction, where MBN does not exceed 400 mV under similar conditions.

At higher plastic strains, the increased density of martensite islands enhances the magnetic exchange interactions among neighbouring islands, resulting in stronger MBN pulses. The number of MBN pulses exceeding the background noise increases with both the plastic strain and strain rate from around 1000 at 5% plastic strain to approximately 11,500 at 60% strain compared to the total number of detected pulses. The reduced MBN intensity at higher strain rates is also attributed to elevated temperatures during deformation, which lowers the susceptibility of AISI 321 to phase transformation. At lower strains, MBN increases primarily due to the growing number of strong pulses, whereas at higher strains, the increasing pulse strength becomes the dominant factor.

Given these findings, the MBN technique provides a fast, non-destructive method for monitoring austenitic steel components to evaluate the presence and volume fraction of strain-induced martensite. Moreover, it serves as a practical tool for optimising deformation parameters to minimise undesirable phase transformations during processing.

## Figures and Tables

**Table 1:** Chemical composition of the austenitic steel AISI 321 in wt. % (according to the standard).

Fe	C	Mn	Cr	Ni	S	P	Si	Ti
bal.	0.08	2	17 ÷ 19	9 ÷ 12	0.03	0.045	1	5 × %C

## Data Availability

The raw data supporting the conclusions of this article will be made available by the authors on request.

## References

[1] Berns H., Gavriljuk V., Riedner S. (2012). High Interstitial Stainless Steels.

[2] Mangonon P.L., Thomas G. (1970). The martensite phases in 304 stainless steel. Metall. Trans..

[3] Mine Y., Horita Z., Murakami Y. (2009). Effect of hydrogen on martensite formation in austenitic stainless steels in high-pressure torsion. Acta Mater..

[4] Shukla S., Patil A.P. (2019). Effect of strain induced martensite reversal on the degree of sensitisation of metastable austenitic stainless steel. Proc. Struct. Integr..

[5] Hotz H., Kirsch B. (2020). Influence of tool properties on thermomechanical load and surface morphology when cryogenically turning metastable austenitic steel AISI 347. J. Manuf. Process..

[6] Talonen J., Aspegren P., Hänninen H. (2004). Comparison of different methods for measuring strain induced α’ martensite content in austenitic steels. Mater. Sci. Technol..

[7] Haušild P., Davydov V., Drahokoupil J., Landa M., Pilvin P. (2010). Characterization of strain-induced martensitic transformation in a metastable stainless steel. Mater. Des..

[8] Haušild P., Kolařík K., Karlík M. (2013). Characterization of strain-induced martensitic transformation in A301 stainless steel by Barkhausen noise measurement. Mater. Des..

[9] Astudilo M.R.N., Nicolás M.N., Ruzzante J., Gómez M.P., Ferrari G.C., Padovese L.R., Pumarega M.I.L. (2015). Correlation between martensitic phase transformation and magnetic Barkhausen noise of AISI 304 steel. Proc. Mater. Sci..

[10] Kleber X., Barroso S.P. (2010). Investigation of shot-peened austenitic stainless steel 304L by means of magnetic Barkhausen noise. Mater. Sci. Eng. A.

[11] Tavares S.S.M., Noris L.F., Pardal J.M., da Silva M.R. (2019). Temper embrittlement of super martensitic stainless steel and non-destructive inspection by magnetic Barkhausen noise. Eng. Fail. Anal..

[12] Jiles D. (2016). Introduction to Magnetism and Magnetic Materials.

[13] Ktena A., Hristoforou E., Gerhardt G.J.L., Missell F.P., Landgraf F.J.G., Rodrigues D.L., Albertis-Campos M. (2014). Barkhausen noise as a microstructure characterisation tool. Phys. B.

[14] Roskosz M., Fryczowski K., Tuz L., Wu J., Schabowicz K., Logoń D. (2021). Analysis of the Possibility of Plastic Deformation Characterisation in X2CrNi18-9 Steel Using Measurements of Electromagnetic Parameters. Materials.

[15] Rydz D., Mróz S., Szota P., Stradomski G., Garstka T., Dyl T.C. (2025). The Analysis of Plastic Forming in the Rolling Process of Difficult-to-Deform Ti + Ni Layered Composites. Materials.

[16] Anglada-Rivera J., Padovese L.R., Capó-Sanchez J. (2001). Magnetic Barkhausen noise and hysteresis loop in commercial carbon steel: Influence of applied tensile stress and grain size. J. Magn. Magn. Mater..

[17] Pitoňák M., Ondruš J., Zgútová K., Neslušan M., Moravec J. (2023). Influence of Strain Rate on Plastic Deformation of the Flange in Steel Road Barrier. Materials.

[18] Batista L., Rabe U., Altpeter I., Hirsekom S., Dobmann G. (2014). On the mechanism of non-destructive evaluation of cementite content in steel using a combination of magnetic Barkhausen noise and magnetic force microscopy techniques. J. Magn. Magn. Mater..

[19] Blažek D., Neslušan M., Mičica M., Pištora J. (2016). Extraction of Barkhausen noise from the measured raw signal in high-frequency regimes. Measurement.

[20] Neslušan M., Haušild P., Šugárová J., Minárik P., Trojan K., Jambor M., Šugár P. (2021). Barkhausen Noise Emission in AISI 321 Austenitic Steel Originating from the Strain-Induced Martensite Transformation. Metals.

[21] Matěj Z., Kadlecová A., Janeček M., Matějová L., Dopita M., Kužel R. (2014). Refining bimodal microstructure of materials with MSTRUCT. Powder Diffr..

[22] Varga R. (2014). Domain Walls and Their Dynamics.

[23] Chikazumi S. (2005). Physics of Ferromagnetism.

[24] Manh T.L., Caleyo F., Hallen J.M., Pérez-Benitez J.A., Espina-Hernández J.H. (2017). Novel method for the accurate determination of magnetocrystalline energy from Barkhausen noise in ferromagnetic materials. Mater. Sci. Eng. B.

[25] He Y., Mehdi M., Hilinski E.J., Edrisy A. (2018). The coarse-process characterisation of local anisotropy on non-oriented electrical steel using magnetic Barkhausen noise. J. Magn. Magn. Mater..

[26] Neslušan M., Bahleda F., Minárik P., Zgútová K., Jambor M. (2019). Non-destructive monitoring of corrosion extent in steel rope wires via Barkhausen noise emission. J. Magn. Magn. Mater..

[27] Neslušan M., Minárik P., Grenčík J., Trojan K., Zgútová K. (2019). Non-destructive evaluation of the railway wheel surface damage after long-term operation via Barkhausen noise technique. Wear.

[28] Cullity B.D., Graham C.D. (2009). Introduction to the Magnetic Materials.

[29] Martínez-Ortiz P., Pérez-Benitez J.A., Espina-Hernández J.H., Caleyo F., Hallen J.M. (2015). On the estimation of the magnetic easy axis in pipeline steels using magnetic Barkhausen noise. J. Magn. Magn. Mater..

[30] Stutius W., Dillinger J.R. (1973). Magnetic and thermal properties of some austenitic stainless steels at low temperatures. J. Appl. Phys..

[31] Brooks J.W., Loretto M.H., Smallman R.E. (1979). In Situ Observations of the Formation of Martensite in Stainless Steel. Acta Metal..

[32] Aristeidakis J.S., Haidemenopoulos G.N. (2022). Constitutive and transformation kinetics modeling of ε-, α -Martensite and mechanical twinning in steels containing austenite. Acta Mater..

[33] Bures R., Neslusan M., Faberova M., Čilliková M., Birčáková Z., Kollar P., Fuzer J., Milyutin V. (2024). Formation of Effective Non-ferromagnetic Barrier in Fe/MgO Soft Magnetic Composite. ACS Appl. Electron. Mater..

[34] Tadić B., Mijatović S., Janićević S., Spasojević D., Rodgers G.J. (2019). The critical Barkhausen avalanches in thin random-field ferromagnets with an open boundary. Sci. Rep..

[35] Neslušan M., Pitoňák M., Minárik P., Tkáč M., Kollár P., Životský O. (2023). Influence of domain walls thickness, density and alignment on Barkhausen noise emission in low alloyed steels. Sci. Rep..

